# A CC-Type Glutaredoxins GRX480 Functions in Cadmium Tolerance by Maintaining Redox Homeostasis in Arabidopsis

**DOI:** 10.3390/ijms252111455

**Published:** 2024-10-25

**Authors:** Ying-Rui Li, Wei Cai, Ya-Xuan Zhang, Ning-Xin Zhang, Qiao-Ling Huang, Ying-Tang Lu, Ting-Ting Yuan

**Affiliations:** 1 State Key Laboratory of Hybrid Rice, College of Life Sciences, Wuhan University, Wuhan 430072, China; 2 Institute of Crop Science of Wuhan Academy of Agriculture Science, Wuhan 430345, China

**Keywords:** cadmium stress, glutaredoxins, GRX480, glutathione, ROS, RCS

## Abstract

Cadmium (Cd) toxicity causes oxidative stress damage in plant cells. Glutaredoxins (GRXs), a type of small oxidoreductase, play a crucial role in modulating thiol redox states. However, whether GRXs act in Cd stress remains to be identified. Here, we reveal that Arabidopsis GRX480, a member of the CC-type family, enhances plant Cd stress tolerance. The *GRX480* mutants exhibit enhanced sensitivity to Cd stress, manifested by shortened root, reduced biomass, lower chlorophyll and proline levels, and decreased photosynthetic efficiency compared with the wild type. The Cd concentration in *GRX480* mutants is higher than the wild type, resulting from the inhibition of Cd efflux and transport genes transcription. Lower levels of GSH were detected in Cd-treated *GRX480* mutants than in the wild type, indicating that *GRX480* regulates plant Cd tolerance by influencing the balance between GSH and GSSG. Furthermore, the hyperaccumulation of reactive oxygen species (ROS) is associated with decreased expression of H_2_O_2_ scavenging genes in Cd-treated *GRX480* mutants. Additionally, more toxic reactive carbonyl species (RCS), produced during oxidative stress, accumulate in Cd-treated *GRX480* mutants than in wild type. Overall, our study establishes a critical role of GRX480 in response to Cd stress, highlighting its multifaceted contributions to detoxification and the maintenance of redox homeostasis.

## 1. Introduction

Human activities such as mining, metal smelting, irrigation with contaminated water, and the use of metal-based fertilizers or agricultural chemicals are associated with Cd accumulation in soil [1,2]. In soils, Cd elements can exist in various forms, and the mobility and availability of Cd in the soil environment are controlled primarily by its adsorption–desorption on the surfaces of soil minerals [3]. As a result, increases in soil pH and the contents of carbonates or organic matter can all enhance the immobilization of Cd in soils and exacerbate soil cadmium contamination [4].

Excessive exposure to Cd leads to its accumulation in plants, which negatively affects plant growth and development, resulting in symptoms such as inhibited growth, chlorosis, and even the death of the entire plant [5,6,7]. Like other heavy metal elements, Cd is highly toxic to plants in their ionic forms and three main mechanisms of Cd have been established. (i) Due to the strong affinity for thiol groups, histidyl- and carboxyl-groups, Cd interacts robustly with proteins and can impair protein functions and (ii) indirectly causes oxidative stress. (iii) Cd^2^⁺ displaces essential cations from specific binding sites, causing functional disruption. For example, Cd inhibits chlorophyll biosynthesis by substitution of Mg^2+^ [8,9,10]. A more pressing concern is that the accumulation of cadmium in plants and its subsequent translocation into the food chain poses a significant threat to human health [8]. Exploring the molecular mechanisms underlying plant responses to Cd stress may lead to the development of new strategies to ensure food security and human health and is therefore of great importance.

Cd toxicity disrupts the absorption and transport of other ions in plant cells, impairs protein and nucleic acid functions, and increases the accumulation of reactive oxygen species (ROS), causing oxidative stress damage [11]. Plants have evolved an array of effective ways to detoxify and tolerate Cd stress [11,12,13,14]. In response to Cd stress, plants reduce the transportation of Cd from roots to stems. Additionally, the root cell walls serve as a barrier to isolate Cd ions and Cd chelates are sequestered within the vacuoles of root cells [15]. Furthermore, plants enhance their antioxidant enzyme activities [16]. In addition to these physical barriers, they also upregulate the synthesis of metallothionein genes or downregulate genes associated with heavy metal transport, which are mechanisms plants employ to combat Cd stress [11]. Plants employ a range of Cd efflux genes, such as *PCR1*, *PCR2*, and *PDR8* [12,17], as well as Cd transport genes including *HMA2*, *HMA3*, and *HMA4* [18,19,20], to reduce Cd accumulation and its long-distance translocation in roots and shoots. Additionally, plants utilize small cysteine-rich molecules like glutathione (GSH) and phytochelatins (PCs) to chelate Cd [21,22]. To counteract Cd-induced ROS and oxidative damage, plants have evolved complex antioxidant systems, enhancing ROS scavenging by activating several antioxidant enzymes: catalase (CAT), superoxide dismutase (SOD) and ascorbate peroxidase (APX) [9]. Glutaredoxins (GRXs), a type of small oxidoreductase that modulates thiol redox states and engages in iron metabolism, play a crucial role in maintaining cellular redox homeostasis [23]. They function as oxidoreductases and work in concert with GSH, glutathione reductase (GR), and NADPH to reduce oxidized disulfide bonds through monothiol or dithiol mechanisms [24]. Notably, while GRXs have been widely shown to play antioxidant roles in bacteria, yeast, and mammals [23,24], their specific functions under Cd stress in plants are not well understood.

In Arabidopsis, there are 31 GRXs, which is a more extensive array than the numbers found in other organisms, such as *Escherichia coli* with 4 and humans with 3 [25]. GRXs are categorized into three classes based on their active site cysteine pair: CPYC-type (class I), CGFS-type (class II), and the most numerous and land plant-specific CC-type GRXs (class III) [26]. The diverse roles of CC-type GRXs (ROXYs) in plant development and stress response have been increasingly recognized. For instance, GRXC7/ROXY1 controls meristematic activity during floral organogenesis by negatively regulating the TGA transcription factor PERIANTHIA (PAN) [27], while GRXC7/ROXY1 and GRXC8/ROXY2 are redundantly required for microsporogenesis through their interaction with TGA9 and TGA10 [28]. GRXC14/ROXY8 and GRXC13/ROXY9 were interacting with TGA1 and TGA4 to negatively regulate auxin-mediated hyponastic growth [29]. In addition to research on plant development, a study has indicated that *GRXS13/ROXY18* can be induced by the herbicide methylviologen (MV) and high light exposure, and the RNAi line accumulates more superoxide anions $O_{2}^{\cdot -}$, suggesting its involvement in oxidative stress response [30]. *GRX480/ROXY19*, a significant CC-type GRX that functions in plant disease resistance, interacts with TGA2, TGA5 and TGA6 and has been shown to negatively regulate plant defense processes by suppressing the transcription of jasmonic acid (JA) and ethylene (ET) response genes [31,32,33]. Furthermore, it has been reported that the transcription of *GRX480/ROXY19* is induced in response to excess light stress [34]. Despite these findings, the role of GRX480 under abiotic stress, particularly its function under heavy metal stress, remains unclear.

Here, our study establishes the role of GRX480 in plant Cd tolerance. Using two T-DNA insertion knock-down mutants, *grx480-1* and *grx480-2*, we found the inhibition of *GRX480* caused the hypersensitive Cd stress, which manifested as the inhibition of photosynthesis and the enhancement of Cd accumulation. As a member of the GRXs, GRX480 has the potential to bind with GSH and form part of the antioxidant system. In our study, *GRX480* regulates plant Cd tolerance by influencing the balance between GSH and GSSG, as well as inhibiting the accumulation of ROS and reactive carbonyl species (RCS). This study not only provides new insights into the molecular mechanism of plant responses to Cd stress but also offers potential genetic resources for cultivating crop varieties with higher Cd tolerance.

## 2. Results

### 2.1. GRX480 Functions in Plant Cd Stress Tolerance

**Figure 1:**
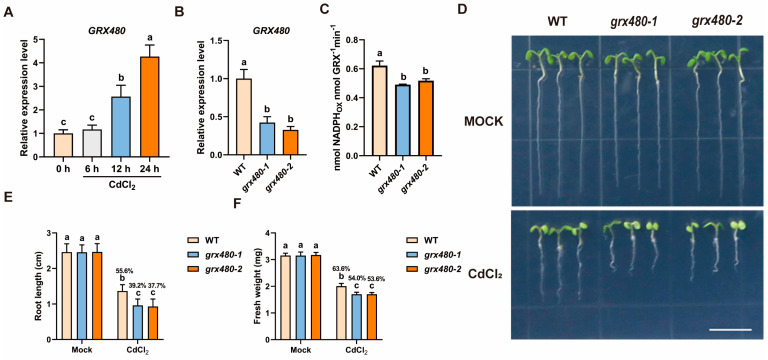
*GRX480* mutants are hypersensitive to Cd stress. (**A**) The expression levels of *GRX480* in 5-day-old wild-type seedlings subjected to 50 µM CdCl_2_ for 0, 6, 12 and 24 h and its control. Values are normalized to the 0 h control, with this baseline set at 1. Values represent the mean ± SEM of three independent experiments. (**B**) The expression levels of *GRX480* in 5-day-old wild-type, *grx480-1* and *grx480-2* seedlings cultivated on 1/2 MS medium. Values are normalized to the untreated WT, with this baseline set at 1. Data are means ± SEM of three independent experiments. (**C**) The HED assay of 5-day-old wild-type, *grx480-1* and *grx480-2* seedlings grown on 1/2 MS medium. Different letters represent significant differences as determined using ANOVA followed by Tukey’s test (*p* < 0.05). (**D**) Representative images of 5-day-old wild-type, *grx480-1* and *grx480-2* seedlings grown on 1/2 MS medium with or without 50 µM CdCl_2_. Bar = 10 mm. (**E**,**F**) Root length (**E**) and fresh weight (**F**) of 5-day-old seedlings grown on 1/2 MS medium with or without 50 µM CdCl_2_. Values represent the mean ± SD of three independent experiments. Different letters represent significant differences as determined using ANOVA followed by Tukey’s test (*p* < 0.05, *n* > 60).

*GRX480*, encoding a protein of the CC-type GRXs family, has not been reported to be involved in Cd stress [35]. To investigate its potential role in plant response to Cd stress, we first assessed the effect of Cd stress on *GRX480* expression. Arabidopsis seedlings grown on 1/2 MS medium were treated with 50 µM CdCl_2_ for 0, 6, 12 and 24 h. RT-qPCR analysis revealed a significant induction of *GRX480* expression with increasing time (Figure 1A). To further explore the function of *GRX480* in Cd stress, we employed two T-DNA insertion mutants of *GRX480*, *grx480-1* and *grx480-2* (Supplemental Figure S1A,B). RT-qPCR analysis revealed that *GRX480* mutants exhibited reduced *GRX480* expression (Figure 1B). Additionally, previous studies on poplar have demonstrated that the in vitro purified simulated CC-type GRXs exhibit oxidoreductase activity [36]. We extracted the total proteins of WT and *GRX480* mutants and detected GRX enzyme activities using the HED assay, and the results indicated that the activities of GRXs decreased in the mutants compared to the wild type (Figure 1C).

Cd, recognized as a highly phytotoxic heavy metal, significantly affects plant growth, development, and a range of physiological processes. Previous studies have demonstrated that a 50 µM CdCl_2_ concentration is effective for investigating plant responses to Cd stress, as it significantly inhibits growth without inducing lethal toxicity [5,6,7]. Accordingly, we cultivated wild-type and *GRX480* mutants on 1/2 MS medium with or without 50 µM CdCl_2_ for 5 days. Under control conditions, the WT and *GRX480* mutants exhibited similar root lengths and fresh weight. However, under Cd stress, the root length and biomass of the *GRX480* mutants were significantly reduced than those of the WT (Figure 1D–F). While the root length of WT was reduced to 55.6 ± 7.18% after Cd treatment compared to the control conditions, the root lengths of the *grx480-1* and *grx480-2* were markedly shorter after Cd treatment, being 39.2 ± 7.19% and 37.7 ± 8.47% of those of the untreated mutants, respectively (Figure 1D,E). Furthermore, fresh weight measurements showed that the biomass of *grx480-1* and *grx480-2* decreased to 54.0 ± 2.37% and 53.6 ± 2.02% after Cd treatment compared to the control conditions, respectively, whereas that of the wild type was reduced to 63.6 ± 3.32% (Figure 1F). Taken together, these results indicated that the knock-down of *GRX480* causes hypersensitivity of plants to Cd stress. This suggests GRX480 is involved in plant Cd tolerance.

### 2.2. Suppressing GRX480 Reduces Plant Cd Tolerance with Decreased Photosynthetic Activity and Proline Levels

**Figure 2:**
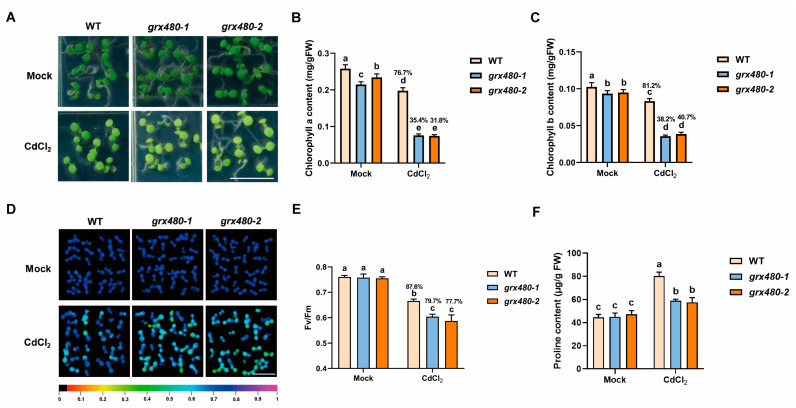
*GRX480* mutants exhibit serious damage in photosynthesis under Cd stress. (**A**–**C**) Representative images (**A**) and chlorophyll a (**B**) and chlorophyll b (**C**) content of 5-day-old wild-type, *grx480-1* and *grx480-2* seedlings cultivated on 1/2 MS medium supplemented with or without 50 µM CdCl_2_. Bar = 10 mm. (**D**,**E**) Representative images (**D**) and intensity (**E**) of *Fv/Fm* of 5-old-day wild-type, *grx480-1* and *grx480-2* seedlings cultivated on 1/2 MS medium with or without 50 µM CdCl_2_. Bar = 10 mm. (**F**) The proline content of 5-day-old wild-type, *grx480-1* and *grx480-2* seedlings cultivated on 1/2 MS medium supplemented with or without 50 µM CdCl_2_. Values represent the mean ± SD of three independent experiments. Different letters represent significant differences as determined using ANOVA followed by Tukey’s test (*p* < 0.05).

The toxicity of Cd to plants also includes damage to chloroplasts [37]. To further explore the functions of GRX480 in plant Cd tolerance, both WT and *GRX480* mutants were planted on 1/2 MS medium with or without 50 µM CdCl_2_. After growing horizontally for 5 days, their growth status was monitored. As shown, *GRX480* mutants displayed significant chlorosis under Cd stress compared to the WT (Figure 2A). The quantitative result exhibited that *GRX480* mutants had lower chlorophyll content compared to the WT (Figure 2B,C). Under Cd stress, the chlorophyll a content of WT was reduced to 76.7 ± 3.08%; however, in the *grx480-1* and *grx480-2*, the content of chlorophyll a plummeted to 35.4 ± 1.71% and 31.8 ± 1.28%, respectively, compared with untreated seedlings (Figure 2B). The quantification of chlorophyll b showed similar results, indicating that Cd stress-mediated reduction in chlorophyll content was aggravated in the *grx480-1* and *grx480-2* mutant (Figure 2C).

Based on the reduction in photosynthetic pigment content, we further evaluated the photosynthetic efficiency of *GRX480* mutants under Cd stress. *Fv/Fm*, representing the maximum photochemical quantum yield of chlorophyll fluorescence, is an important indicator for assessing the photosynthetic potential [38]. Measurements of *Fv/Fm* showed that Cd stress resulted in a reduction in light energy conversion efficiency of the Photosystem II (PSII), with the *Fv/Fm* of WT decreased to 87.6 ± 0.92% after Cd treatment compared with untreated. However, *grx480-1* and *grx480-2* mutants showed further declines to 79.7 ± 1.22% and 77.7% ± 3.11% compared to untreated seedlings, respectively, suggesting *GRX480* may stabilize photosynthesis under Cd stress (Figure 2D,E).

The accumulation of proline content is associated with an adaptive response of plants to adverse stress, such as salinity, drought, and heavy metal exposure [39,40,41]. Here, the proline content with and without Cd stress was examined. Under control conditions, the proline content of WT and *GRX480* mutants was similar. However, under Cd treatment conditions, while the proline content of WT accumulated to 80.4 ± 3.21 µg/g FW, the proline content of *grx480-1* and *grx480-2* was 58.9 ± 1.39 µg/g FW, 57.5 ± 4.09 µg/g FW, respectively (Figure 2F). Notably, proline accumulation was significantly lower in *GRX480* mutants than in the wild type under Cd stress. These results suggest that the inhibition of *GRX480* suppresses plant Cd tolerance leading to lowering photosynthetic activity and proline levels.

### 2.3. GRX480 Mutants Alter Cd Accumulation by Modulating Cd Efflux and Transport Genes Transcription

**Figure 3:**
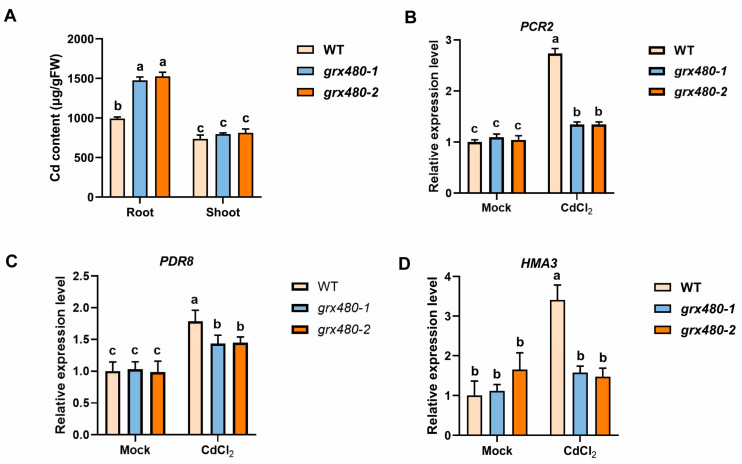
More cadmium has accumulated in *GRX480* mutants. (**A**) Cd contents in the root and shoot of 10-old-day wild-type, *grx480-1* and *grx480-2* seedlings cultivated on 1/2 MS medium supplemented with or without 50 µM CdCl_2_. Values represent the mean ± SD of three independent experiments. (**B**–**D**) The expression levels of *PCR2* (**B**), *PDR8* (**C**) and *HMA3* (**D**) of 5-day-old wild-type, *grx480-1* and *grx480-2* seedlings cultivated on 1/2 MS medium supplemented with or without 50 µM CdCl_2_. The expression of these genes was quantified by RT-qPCR and the values are normalized to the untreated WT, with this baseline set at 1. Values represent the mean ± SEM of three independent experiments. Different letters represent significant differences as calculated using ANOVA followed by Tukey’s test (*p* < 0.05).

To assess whether GRX480 affects Cd accumulation under Cd stress, we harvested roots and shoot tissue from WT and *GRX480* mutant seedlings that were grown on 1/2 MS medium supplemented with 50 µM CdCl_2_, and subsequently measured the Cd content. We found that *GRX480* mutants accumulated more Cd than the WT in the roots (Figure 3A). In the shoots, although a slight increase was detected in the *grx480-1* and *grx480-2* mutants, no significant differences were statistically analyzed between the *GRX480* mutants and the wild type under Cd stress (Figure 3A). It means that GRX480 likely influences Cd accumulation in roots, contributing to plant tolerance to Cd.

Metal ions are typically absorbed by active transport by plant root cells. Numerous studies have been conducted on the absorption, transport, and efflux of Cd ions [2,12,17,18,42]. In this study, we examined the expression of Cd efflux and transport-related genes in *GRX480* mutants. Under Cd stress, the transcription of *PCR2* and *PDR8* was induced in WT and *GRX480* mutants; however, the induction of these genes by Cd stress was alleviated in *GRX480* mutants (Figure 3B,C). Notably, *HMA3*, which mediates vacuolar sequestration of Cd, exhibited similar expression levels in both WT and *GRX480* mutants under control conditions; however, its induction by Cd stress was observed in WT, while this induction was abolished in *GRX480* mutants (Figure 3D). These results suggest that the increased accumulation of Cd in mutants is derived from the alteration of Cd efflux and transport. These findings could potentially account for the observed difference in Cd content between the roots and shoots of the WT and *GRX480* mutants.

### 2.4. GRX480 Influences the Balance Between GSH and GSSG During Plant Response to Cd Stress

**Figure 4:**
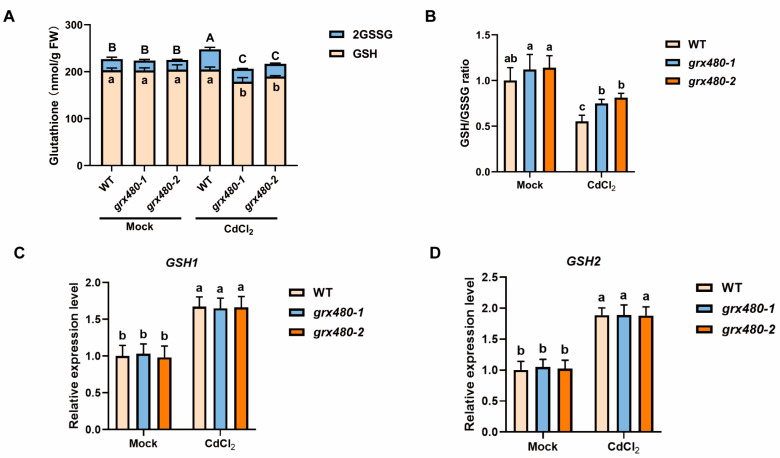
*GRX480* mutants have less GSH compared with WT. (**A**) Glutathione (GSH plus 2GSSG) content of 5-day-old wild-type, *grx480-1* and *grx480-2* seedlings cultivated on 1/2 MS medium supplemented with or without 50 µM CdCl_2_. Values represent the mean ± SD of three independent experiments. Different letters represent significant differences as determined using ANOVA followed by Tukey’s test (*p* < 0.05), and capital letters represent total glutathione statistical analysis, while lowercase letters represent GSH difference analysis. (**B**) The relative ratio of GSH/GSSG of 5-day-old wild-type, *grx480-1* and *grx480-2* seedlings cultivated on 1/2 MS medium supplemented with or without 50 µM CdCl_2_. The data of untreated WT were set to 1. Values represent the mean ± SD of three independent experiments. Different letters represent significant differences as determined using ANOVA followed by Tukey’s test (*p* < 0.05). (**C**,**D**) The expression levels of *GSH1* (**C**) and *GSH2* (**D**) of 5-day-old wild-type, *grx480-1* and *grx480-2* seedlings cultivated on 1/2 MS medium supplemented with or without 50 µM CdCl_2_. The expression of these genes was quantified by RT-qPCR and the values are normalized to the untreated WT, with this baseline set at 1. Values represent mean ± SEM of three independent experiments. Different letters represent significant differences as calculated utilizing ANOVA followed by Tukey’s test (*p* < 0.05).

Given that GRX480, as a member of the GRXs, has the potential to bind with GSH and form the antioxidant system, acting as an electron donor in the reduction of disulfide bonds [35]. We hypothesize that the functional alterations of GRX480 may affect the levels of GSH and consequently impact the Cd tolerance of plants. Subsequently, we measured glutathione content, including reduced GSH and oxidized GSSG, in WT and *GRX480* mutants treated with or without 50 µM CdCl_2_. We found that the content of GSH was increased and oxidized GSSG was unchanged, leading to inhibition of GSH/GSSH ratio, in the wild type under Cd stress compared with untreated plants (Figure 4A,B). However, in Cd treated-*GRX480* mutants, total glutathione is reduced accompanied by a significant decrease in GSH content compared with the wild type under Cd stress, indicating that GRX480 regulates the response of plants to Cd by inhibiting GSH, leading to influencing the balance between GSH and GSSG (Figure 4A,B). Besides, to investigate whether the altered GSH content is due to an impact on GSH synthesis, we assessed *GSH1* and *GSH2* expression levels using RT-qPCR, which are coding two glutathione synthetase and induced by Cd stress [21]. Our results revealed no significant differences between WT and mutants detected both under control and Cd stress (Figure 4C,D), indicating that GRX480 regulates the balance between GSH and GSSG rather than directly impacting the synthesis of GSH. In conclusion, our results reveal that the knock-down of *GRX480* contributes to the hypersensitivity phenotype to Cd stress through the breaking of the balance between GSH and GSSG.

### 2.5. GRX480 Regulates ROS Homeostasis Under Cd Stress

It is reported that Cd stress causes the production of ROS, leading to oxidative damage. Through 3,3-diaminobenzidine (DAB) staining and nitroblue tetrazolium (NBT) staining, we examined the levels of H_2_O_2_ and $O_{2}^{\cdot -}$ in WT and *GRX480* mutants.

**Figure 5:**
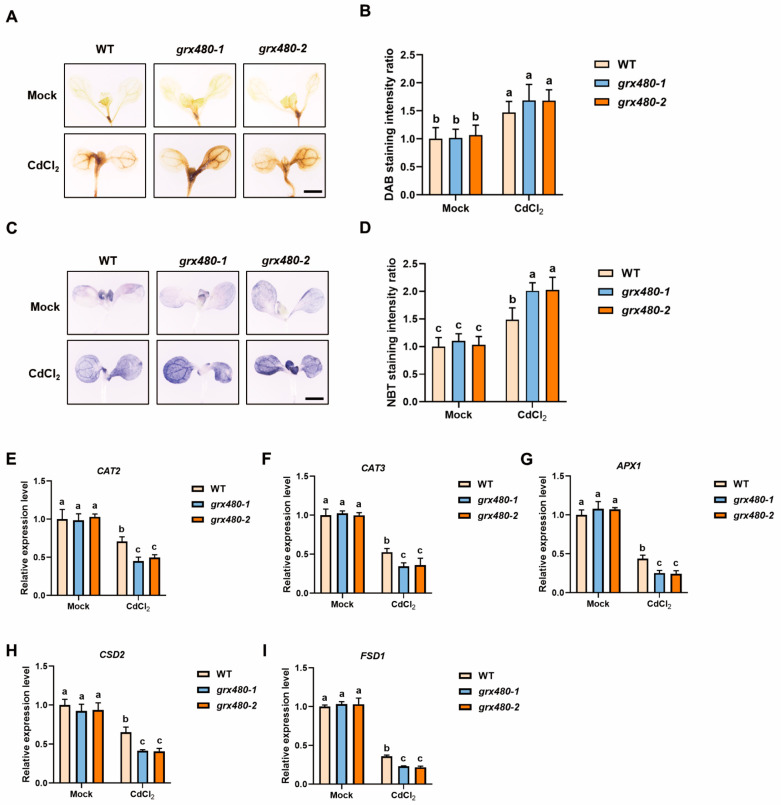
The homeostasis of ROS is disrupted in *GRX480* mutants. (**A**) Representative DAB-staining images of leaves from 5-day-old wild-type, *grx480-1* and *grx480-2* seedlings cultivated on 1/2 MS medium supplemented with or without 50 µM CdCl_2_. Bar = 1000 µm. (**B**) The relative intensity in (**A**). The data of untreated WT were set to 1. (**C**) Representative NBT staining images of leaves from 5-day-old wild-type, *grx480-1* and *grx480-2* seedlings cultivated on 1/2 MS medium supplemented with or without 50 µM CdCl_2_. Bar = 1000 µm. (**D**) The relative intensity in (**C**). The data of untreated WT were set to 1. Values represent the mean ± SD of three independent experiments. Different letters represent significant differences as determined using ANOVA followed by Tukey’s test (*p* < 0.05 *n* > 30). (**E**–**I**) The expression levels of *CAT2* (**E**), *CAT3* (**F**), *APX1* (**G**), *CSD2* (**H**) and *FSD1* (**I**) of 5-day-old wild-type, *grx480-1* and *grx480-2* seedlings cultivated on 1/2 MS medium supplemented with or without 50 µM CdCl_2_. The expression of these genes was quantified by RT-qPCR and the values are normalized to the untreated WT, with this baseline set at 1. Values represent mean ± SEM of three independent experiments. Different letters represent significant differences as calculated utilizing ANOVA followed by Tukey’s test (*p* < 0.05).

Under Cd stress, both WT and *GRX480* mutants exhibited an increase in the precipitation of colored substances formed by DAB and NBT staining, with the *grx480-1* and *grx480-2* showing a more pronounced enhancement (Figure 5A,B). We quantified the staining intensity of DAB and NBT, although there was no statistically significant difference in the DAB staining intensity between WT and *GRX480* mutants (Figure 5C,D).

To elucidate the molecular mechanism of GRX480-mediated ROS homeostasis changes under Cd stress, we employed the RT-qPCR assay to measure the transcription levels of several genes that encode the antioxidant enzymes, such as *CAT2* and *CAT3* (encoding two catalases), *APX1* (encoding a cytosolic ascorbate peroxidase); *CSD2* and *FSD1* (encoding two superoxide dismutases). As shown, Cd treatment markedly suppressed the expression of all these genes in WT and *GRX480* mutants, notably, the inhibition of these genes was aggravated in *GRX480* mutants (Figure 5E–I). These results indicate that the *GRX480* regulates ROS homeostasis under Cd stress.

### 2.6. GRX480 Mutants Show the Hyperaccumulation of Toxic RCS Under Cd Stress

**Figure 6:**
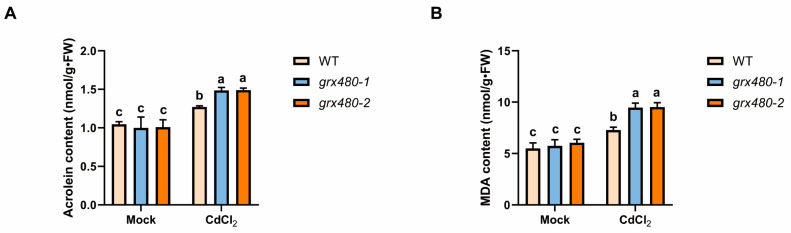
More RCS has accumulated in *grx480* mutants. (**A**,**B**) The content of acrolein (**A**) and MDA (**B**) in 5-day-old wild-type, *grx480-1* and *grx480-2* seedlings cultivated on 1/2 MS medium supplemented with or without 50 µM CdCl_2_. Values represent the mean ± SD of three independent experiments. Different letters represent significant differences as determined utilizing ANOVA followed by Tukey’s test (*p* < 0.05).

This oxidative stress often leads to intracellular lipid peroxidation, resulting in the production of a large amount of toxic reactive carbonyl species (RCS) [43]. *GRX480* was thought to be involved in the detoxification process of RCS under photooxidative stress, but its function under Cd stress is still unknown [34,44]. Consequently, we employed LC-MS to examine the accumulation of RCS in WT and *GRX480* mutants under Cd stress. Under Cd stress, two toxic reactive carbonyl species (RCS), acrolein and malondialdehyde (MDA), were significantly induced in both WT and *GRX480* mutants. Notably, *grx480-1* and *grx480-2* accumulated more acrolein and malondialdehyde (MDA) than the WT under Cd stress (Figure 6A,B). These findings suggest that GRX480 alleviate oxidative stress in plants by detoxifying RCS, thereby conferring Cd tolerance.

### 2.7. Overexpression of GRX480 Enhances Plant Tolerance to Cd Stress

**Figure 7:**
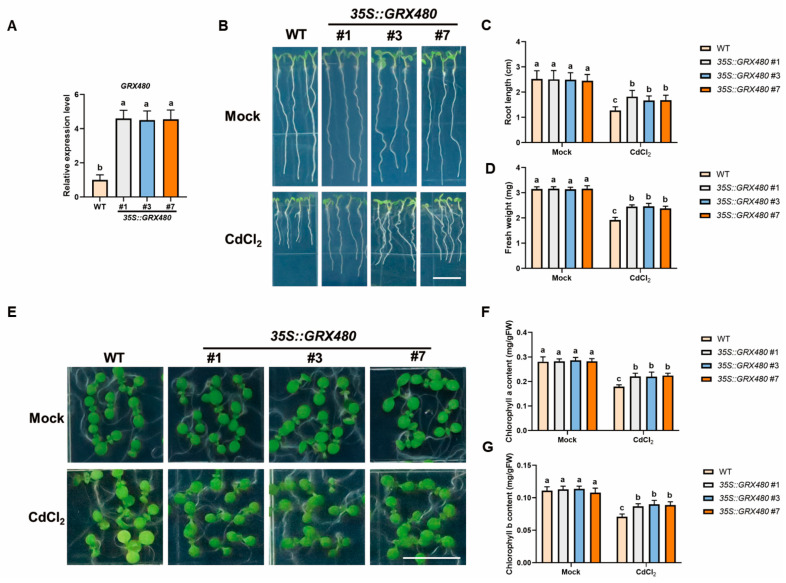
Overexpression of *GRX480* enhances Cd stress tolerance. (**A**) The expression levels of *GRX480* in 5-day-old wild-type, *35S::GRX480* #1, #3 and #7 seedlings cultivated on 1/2 MS medium. Values are normalized to the WT, with this baseline set at 1. Values represent the mean ± SEM of three independent experiments. Different letters represent significant differences as determined using ANOVA followed by Tukey’s test (*p* < 0.05). (**B**) Representative images of 5-day-old wild-type, *35S::GRX480* #1, #3 and #7 seedlings cultivated on 1/2 MS medium with or without 50 µM CdCl_2_. Bar = 10 mm. (**C**,**D**) Root length (**C**) and fresh weight (**D**) of 5-day-old seedlings grown on 1/2 MS medium with or without 50 µM CdCl_2_. Values represent the mean ± SD of three independent experiments. Different letters represent significant differences as determined using ANOVA followed by Tukey’s test (*p* < 0.05, *n* > 60). (**E**–**G**) Representative images (**E**) and chlorophyll a (**F**) and chlorophyll b (**G**) content of 5-day-old wild-type, *35S::GRX480* #1, #3 and #7 seedlings cultivated on 1/2 MS medium supplemented with or without 50 µM CdCl_2_. Bar = 10 mm. Values represent the mean ± SD of three independent experiments. Different letters represent significant differences as determined using ANOVA followed by Tukey’s test (*p* < 0.05).

To further explore whether the overexpression of *GRX480* enhances plant tolerance to Cd stress, we generated transgenic lines *35S::GRX480* by overexpressing *GRX480* under the control of 35S promoter. RT-qPCR assay revealed that the *GRX480* transcription level in *35S::GRX480* plants was higher than that in WT (Figure 7A). Under normal grown conditions, *35S::GRX480* plants showed similar root length and fresh weight compared to WT. However, under Cd stress, the root length and biomass of the *35S::GRX480* plants were significantly increased compared to those of the WT (Figure 7B–D). In addition, we monitored the effects of chloroplast damage caused by Cd stress in WT and *35S::GRX480* plants cultured horizontally on 1/2MS medium, with or without 50 µM CdCl_2_. As shown, *35S::GRX480* plants displayed significantly reduced chlorosis under Cd stress compared with the WT (Figure 7E). The quantitative result exhibited that *35S::GRX480* had higher chlorophyll content compared to the WT (Figure 7F,G). In summary, *GRX480* overexpression enhanced plant tolerance to Cd stress, consistent with the hypersensitive phenotype of the *grx480* mutants to Cd stress, further confirming the positive role of *GRX480* in plant tolerance to Cd stress.

## 3. Discussion

Glutaredoxins (GRXs), small and ubiquitous thiol reductases within the thioredoxins (TRXs) superfamily, and most of the biochemical and structural studies on plant GRXs have been focused on Class I and Class II isoforms. They utilize GSH as a cofactor to form an antioxidant system and serve as an electron donor, which is essential for plant growth, development, and response to oxidative stress [45,46,47,48,49]. CC-type GRXs are a unique class of GRXs found in land plants [26]. Although as a subfamily of GRXs, it remains unclear whether they possess oxidoreductase activities in vivo [35], previous studies on poplar have demonstrated that the purified simulated CC-type GRXs exhibit oxidoreductase activity in vitro [36]. In our study, we made the discovery that GRX480 plays a crucial role in plant responses to Cd stress by using *GRX480* mutants. We also measured the overall GRX enzyme activity by HED analysis and found that the overall GRX enzyme activity is significantly inhibited in *grx480-1* and *grx480-2*. The enzyme activity of GRXs is associated with its potential to bind with GSH and together with GR, NADPH, etc., forming an antioxidant system as an electron donor in the reduction of disulfide bonds [50]. Furthermore, GSH has been shown to play crucial roles in the response of plants to Cd, as increased accumulation of GSH can enhance Cd tolerance in transgenic lines [21]. Consistently, total glutathione is reduced accompanied by a significant decrease in GSH content in Cd treated-*GRX480* mutants, which are hypersensitive to Cd stress with hyperaccumulation of Cd in roots, compared with the wild type, indicating that GRX480 regulates plant Cd tolerance by influencing the balance between GSH and GSSG.

In order to deal with Cd stress, plants develop effective mechanisms against Cd stress from uptake, transport, compartmentalisation and efflux of Cd. PCR2 is a homolog of the plasma membrane-localized Cys-rich protein PCR1, which mediates the efflux of Cd and confers plant Cd tolerance [17,42]. PDR8, belonging to the pleiotropic drug resistance (PDR) subfamily of ABC transporters, is located in the plasma membrane and the root epidermal cells. It plays a role in Cd tolerance by pumping out Cd^2+^ from the plasma membrane of root epidermal cells [12]. HMA3, a P1B-type ATPase located in the vacuolar membrane, is involved in the sequestration of Cd in the vacuolar [18], and genome-wide association studies identify that the *HMA3* gene is the only major locus that causes variation in Cd accumulation in the leaves of Arabidopsis thaliana [51]. Our results demonstrate that the expression of these Cd response genes was suppressed in *GRX480* mutants under Cd stress, potentially contributing to the higher accumulation of Cd content in plants. This suggests that GRX480 plays a role in regulating Cd efflux and transport, thereby enhancing plant tolerance to Cd.

Cd stress triggers the production of ROS in plants, which can cause damage to essential macro molecules like DNA, proteins, and membrane lipids, consequently impairing growth [9]. An array of antioxidant systems has been evolved by plants to scavenge ROS. The efficacy of the antioxidant systems is mirrored by the expression and activity of these antioxidant enzymes. Usually, disruption of their normal expression or activity correlates with exacerbated ROS accumulation [52]. In Arabidopsis, CAT1, CAT2 and CAT3 serve as catalases that decompose H_2_O_2_, especially when H_2_O_2_ is present in relatively high amounts [53,54]. *APX1*, which encodes a cytosolic ascorbate peroxidase, facilitates the conversion of H_2_O_2_ into H_2_O and O_2_ by utilizing ascorbate as a specific electron donor [55]. *CSD2* and *FSD1*, encoding two superoxide dismutases, can detoxify superoxide radicals [56,57]. In our study, the transcription levels of *CAT2*, *CAT3*, *APX1*, *CSD2* and *FSD1* were inhibited after Cd treatment, and this inhibition was more pronounced in *GRX480* mutants than in WT, which was consistent with the observed hyperaccumulation of ROS in mutants. These results suggest that repression of ROS scavenge genes may be the main reason for the hyperaccumulation of ROS in Cd-treated *GRX480* mutants.

For plants, photosynthesis is necessary for their growth and development, and it is similarly essential for agricultural productivity. During heavy metal stress, the chloroplasts of plant cells were damaged, accompanied by the chlorosis of leaves. In our study, we measured the *Fv/Fm*, which assesses the photosynthetic potential [38] by detecting the maximum photochemical quantum yield of chlorophyll fluorescence reflecting the potential of photosynthesis, and found that Cd stress reduced the efficiency of photosynthesis in WT and *GRX480* mutants, and this reduction was further aggravated in *GRX480* mutants. Excess light is a common stressor that affects the activity of photosynthesis, leading to the production of a large amount of toxic reactive carbonyl species (RCS) under excessive light, which causes severe photo-oxidative damage [58]. *GRX480* has been reported to be induced in response to excess light stress and is involved in the detoxification response of RCS in the SCARECROW-LIKE (SCL) 14-dependent protective mechanism [34,44]. However, the role of RCS in Cd stress still requires further investigation. We used LC-MS to detect the levels of two important RCSs: acrolein and MDA. The results showed that Cd caused an accumulation of RCS content in both the WT and *GRX480* mutants but to a greater extent in *grx480-1* and *grx480-2*. This suggests that the reduction in photosynthetic efficiency under Cd stress may be due to the accumulation of RCS, and that GRX480 may play a role in this process.

**Figure 8:**
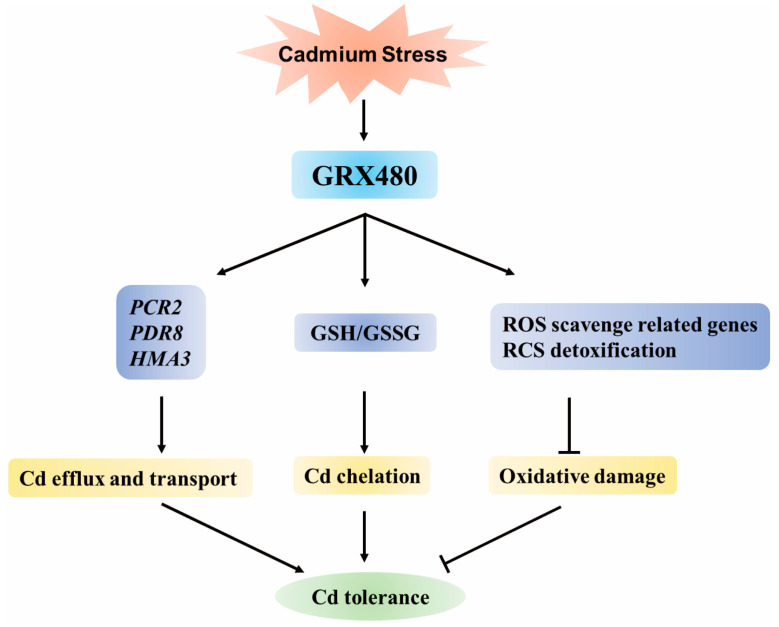
Model for the role of GRX480 in Cd stress tolerance. The Cd-induced *GRX480* is involved in regulating the expression of Cd response genes (*PCR2*, *PDR8*, and *HMA3*) and altering the GSH level to protect plants against Cd stress. The GRX480 mediates the expression of genes involved in scavenging ROS and detoxifying RCS in response to Cd-induced oxidative damage, thereby conferring tolerance to Cd in plants.

In summary, our study reports the crucial role of CC-Type GRX480 in Cd stress and elucidates its involvement in plant detoxification of Cd accumulation. Firstly, GRX480 regulates the expression of genes related to the Cd efflux and transport. Secondly, it influences the content of GSH. Simultaneously, it reduces the oxidative damage caused by heavy metal exposure by regulating the expression of ROS-scavenging genes and detoxing RCS (Figure 8). Our work provides novel insights for understanding plant GRXs.

## 4. Materials and Methods

### 4.1. Plant Materials and Growth Conditions

*Arabidopsis thaliana* ecotype Columbia-0 was used in this study. Two T-DNA insertion lines SALK_031817C and SALK_052093C were obtained from the Arashare (https://www.arashare.cn/index/, accessed on 2 November 2022), a non-profit Arabidopsis Share Center, and verified by PCR, and then they were named *grx480-1* and *grx480-2*, respectively. Primers used for genotyping are listed in Supplemental Table S1.

The seeds were sterilized by surface treatment with 5% (*w*/*v*) bleach for 5 min, followed by three washes with sterile ddH_2_O. Afterwards, the seeds were incubated at 4 °C in the dark for 72 h before being transferred to plates containing half-strength Murashige and Skoog (1/2 MS) medium at pH 5.8, supplemented with 1% (*w*/*v*) agar and 1% (*w*/*v*) sucrose. Seedlings were grown vertically at 23 °C under a 16-h light (100 µmol m^−2^ s^−1^ illumination)/8-h dark photoperiod. For Cd treatment, the medium was supplemented with or without 50 µM CdCl_2_, as previous studies have demonstrated that this concentration is effective for investigating plant responses to Cd stress, significantly inhibiting growth without inducing lethal toxicity [6].

### 4.2. Transformation of Arabidopsis

To generate 35S::GRX480, the CDS of GRX480 was amplified and cloned into pCAMBIA1300 under the control of the 35S promoter. The resultant plasmids were transformed into the wild-type Arabidopsis Col-0 by *Agrobacterium tumefaciens* strain pGV3101 using the florap dip method. Primers used for molecular cloning are listed in Table S1.

### 4.3. Determination of Proline Content

L-proline was utilized as the standard to ascertain proline content, according to the method already documented [7]. In brief, after collection and weight measurement, the leaves were extracted in 3% sulfosalicylic acid. Incubation was performed on 2 mL aliquots of each extract, each mixed with 2 mL of ninhydrin reagent (2.5% [*w*/*v*] ninhydrin, 60% [*v*/*v*] glacial acetic acid, and 40% [*v*/*v*] 6 M phosphoric acid) and 2 mL of glacial acetic acid. The mixture was boiled at 100 °C for 40 min, after which the reaction was discontinued on ice. The mixture was added to toluene (5 mL), vortexed to homogeneity, and incubated at 23 °C for 24 h before the absorbance at 520 nm was recorded using a UV-5200 spectrophotometer (METASH, Shanghai, China).

### 4.4. Measurement of Chlorophyll

Approximately 0.1 g leaves were collected to extract total chlorophyll using 3 mL of 80% (*v*/*v*) acetone at 4 °C for 24 h in darkness. The absorbances of the extract at 663 and 645 nm were measured using spectrophotometry. Chlorophyll contents were calculated as previously described [6].

### 4.5. Measurement of Fv/Fm

The assessment of *Fv/Fm* was carried out according to a method previously applied [59]. First, 5-day-old seedlings treated with or without 50 µM CdCl_2_ were transferred to a dark room at 23 °C for 30 min. A fluorometer HEXAGON-IMAGING-PAM (WALZ, Zealquest, Shanghai, China) was employed to measure *Fv/Fm*.

### 4.6. GRXs Activity Analysis (HED Assay)

Plant proteins were extracted with a buffer containing 375 mM NaCl, 2.5 mM EDTA, 1% β-mercaptoethanol, 125 mM Tris–HCl (pH8.0), and 1% SDS. Crude protein extract concentrations were ascertained by employing the Bradford method. The HED assay was carried out following the method previously described [60]. A mixture of 1 mM GSH, 0.2 mM NADPH, 2 mM EDTA, 0.1 mg/mL bovine serum albumin (BSA), and 6 µg/mL yeast glutathione reductase was prepared in 100 mM Tris-HCl (pH 7.9). Then, 2-Hydroxyethyl disulfide (HED) was added at a final concentration of 0.7 mM. The mixture was vortexed to homogeneity, incubated in 3 min, and protein extracts were added. The decrease in absorbance at 340 nm was followed using a spectrophotometer. Enzyme activities were expressed as micromoles of NADPH oxidized/min.

### 4.7. Determination of Cd Content

Inductively coupled plasma mass spectroscopy (ICP-MS) was employed to determine Cd content according to previously described [5]. Briefly, the roots and shoots of 10-day-old seedlings grown on 1/2 MS medium containing 50 µM CdCl_2_ were harvested separately. Subsequently, about 0.2 g of dried plant materials was ground to a fine powder. Then, it was digested in a mixture of concentrated HNO_3_ and HClO_4_ (4:1, *v*/*v*). The volume of the sample was adjusted to 50 mL using ddH_2_O. Then, the concentration of Cd was detected by ICP-MS.

### 4.8. Determination of Glutathione

Five-day-old seedlings grown on 1/2 MS media with or without 50 µM CdCl_2_ were harvested to measure GSH/GSSG levels according to a previous report [5]. Briefly, glutathione reductase (GR) catalyzes the reduction in GSSG to GSH. Subsequently, GSH reacts with 5,5′-dithiobis-(2-nitrobenzoic acid) (DNTB) resulting in the formation of the yellow-colored compound 2-nitro-5-thiobenzoic acid (TNB) and GSSG. The absorbance at 412 nm can be measured to calculate the total glutathione content. By eliminating GSH, the aforementioned reaction can be used to calculate the GSSG content. The GSH content can then be determined by excluding the GSSG content from the total glutathione content.

### 4.9. LC-MS Analysis of RCS Content

The method was employed as previously described with slight modifications [61]. In summary, 5-day-old seedlings were harvested and extracted by RCS extraction buffer [acetonitrile with 5 µM 2-ethylhexanal (as internal standard) and 0.005% (*w*/*v*) of butylated hydroxytoluene]. Next, the extracts were incubated at 60 °C for 50 min and then centrifuged for 20 min at 27,900× *g*. The supernatant was transferred to a fresh tube, and 0.5 mM of 2,4-DNPH and 0.5 M formic acid were added. The mixture was incubated at 25 °C for 1 h for derivatization. Before injection, the sample was diluted with ultra-pure H_2_O at a ratio of 50:50.

Acrolein-DNPH and malondialdehyde-DNPH were identified and quantified by LC-MS mass spectrometer (Q-Exactive, Thermo Scientific, Waltham, MA, USA) in negative ion mode. A reversed-phase column (Thermo Scientific Hyper GOLD^TM^ C18 100 × 2.1 mm, 3 µm) was selected to separate carbonyl-DNPH compounds. A multistep gradient was used with 0.1% formic acid (*v*/*v*) (A) and acetonitrile (B). The flow rate was maintained at 0.5 mL/min for the entire duration of the 10 min run: 0–2 min, 5% B; 2–7 min, 5%–95% B; 7–9 min, 95% B; 9–10 min, 5% B (all percentages in *v*/*v*). The LC-MS instrument parameters were set as follows: capillary temperature at 320 °C, spray voltage at 3.2 kV, sheath gas flow rate at 40, auxiliary gas flow rate at 15, and S-lens radio frequency level at 55. Full MS mode was employed to obtain the mass spectra, with a resolution setting of 70,000 at full width at half maximum.

### 4.10. RNA Extraction and RT-qPCR Analysis

The RNA was extracted following the method previously described [62]. In brief, plants were flash-frozen in liquid nitrogen and ground to a fine powder. TRIzol reagent (Invitrogen, Carlsbad, CA, USA) was used to extract RNA and RQ1 RNase-free DNase I (Promega, Madison, WI, USA) was employed to remove DNA. RNA reverse transcription was conducted using the ReverTra Ace kit (TOYOBO, Tsuruga-shi, Japan) as per the instructions. qPCR was carried out on BioRad CFX96 instrument (Hercules, CA, USA) with SYBR Green I dye (Invitrogen), following a program of 95 °C for 3 min, 40 cycles of 95 °C for 15 s, and 60 °C for 45 s. Reference genes selected for the study included *Protein Phosphatase 2A Subunit A3* (*PP2AA3*, AT1G13320) and *Eukaryotic Translation Initiation Factor 4A1* (*EIF4A1*, AT3G13920). Each experiment was conducted with three separate biological replicates and three technical replicates. Primer sequences are listed in Supplemental Table S1.

### 4.11. DAB and NBT Staining

The 5-day-old seedlings were harvested to determine H_2_O_2_ and $O_{2}^{\cdot -}$ accumulation as previously described [63,64]. Incubation of the seedlings was carried out using fresh prepared DAB staining solution [1 mg/mL DAB, 10 mM Na_2_HPO_4_ and 0.1% (*v*/*v*) Tween-20] and NBT staining solution [0.1 mg/mL NBT in 25 mM HEPES buffer (pH 7.6)] for 8 and 2 h, respectively. Subsequently, chlorophyll was removed using 70% (*v*/*v*) ethanol. Stereomicroscopy was employed for photography, and the staining intensity was statistically analyzed using ImageJ version 1.54k.

### 4.12. Statistical Analysis

All experiments were performed with at least three repetitions. The significance of differences was determined by ANOVA, as indicated in the figure legends.

## 5. Conclusions

In conclusion, our research revealed the pivotal role of GRX480 in modulating plant responses to Cd stress, highlighting its multifaceted regulatory capabilities in enhancing plant Cd tolerance. The Cd-induced GRX480 enhances plants’ Cd tolerance by regulating the expression of Cd efflux and transport gene transcription to reduce Cd enrichment, as well as the expression of ROS scavenge enzyme encoding genes to reduce oxidative damage. Moreover, as a member of glutaredoxins, GRX480 is involved in influencing the balance between GSH and GSSG, thereby conferring tolerance to Cd in plants.

CC-type class GRXs are widely present in land plants. However, less research focuses on their roles in plant development and stress tolerance. Therefore, the role of *GRX480* homologous genes in other plant species, especially crops, in enhancing plant Cd tolerance is worth further exploring. These results will be used for the crop breeding design for high Cd stress tolerance and low cadmium enrichment germplasm resources, and ultimately serve food security and human health.

## Data Availability

The data are present within the article or the Supplementary Materials.

## Supplementary Materials

The supporting information can be downloaded at: https://www.mdpi.com/article/10.3390/ijms252111455/s1.

## References

[1] da Rosa Couto R., Faversani J., Ceretta C.A., Ferreira P.A.A., Marchezan C., Basso Facco D., Garlet L.P., Silva J.S., Comin J.J., Bizzi C.A. (2018). Health risk assessment and soil and plant heavy metal and bromine contents in field plots after ten years of organic and mineral fertilization. Ecotoxicol. Environ. Saf..

[2] Zhao F.J., Tang Z., Song J.J., Huang X.Y., Wang P. (2022). Toxic metals and metalloids: Uptake, transport, detoxification, phytoremediation, and crop improvement for safer food. Mol. Plant.

[3] Hu Y., Cheng H., Tao S. (2016). The Challenges and Solutions for Cadmium-contaminated Rice in China: A Critical Review. Environ. Int..

[4] Hussain B., Ashraf M.N., Shafeeq Ur R., Abbas A., Li J., Farooq M. (2021). Cadmium stress in paddy fields: Effects of soil conditions and remediation strategies. Sci. Total Environ..

[5] Zhang B.-L., Guo C.-C., Ding F., Lu Y.-T., Fu Z.-W. (2019). 14-3-3s function in plant cadmium response by changes of glutathione and glutathione synthesis in Arabidopsis. Environ. Exp. Bot..

[6] Zhang Q., Cai W., Ji T.T., Ye L., Lu Y.T., Yuan T.T. (2020). WRKY13 Enhances Cadmium Tolerance by Promoting D-CYSTEINE DESULFHYDRASE and Hydrogen Sulfide Production. Plant Physiol..

[7] Zhu J., Wang W.S., Ma D., Zhang L.Y., Ren F., Yuan T.T. (2016). A role for CK2 beta subunit 4 in the regulation of plant growth, cadmium accumulation and H(2)O(2) content under cadmium stress in Arabidopsis thaliana. Plant Physiol. Biochem..

[8] Clemens S., Ma J.F. (2016). Toxic Heavy Metal and Metalloid Accumulation in Crop Plants and Foods. Annu. Rev. Plant Biol..

[9] Sharma S.S., Dietz K.J. (2009). The relationship between metal toxicity and cellular redox imbalance. Trends Plant Sci..

[10] Wang Y., Jiang X., Li K., Wu M., Zhang R., Zhang L., Chen G. (2014). Photosynthetic responses of *Oryza sativa* L. seedlings to cadmium stress: Physiological, biochemical and ultrastructural analyses. Biometals.

[11] Clemens S., Aarts M.G., Thomine S., Verbruggen N. (2013). Plant science: The key to preventing slow cadmium poisoning. Trends Plant Sci..

[12] Kim D.Y., Bovet L., Maeshima M., Martinoia E., Lee Y. (2007). The ABC transporter AtPDR8 is a cadmium extrusion pump conferring heavy metal resistance. Plant J..

[13] Peng J.S., Wang Y.J., Ding G., Ma H.L., Zhang Y.J., Gong J.M. (2017). A Pivotal Role of Cell Wall in Cadmium Accumulation in the Crassulaceae hyperaccumulator Sedum plumbizincicola. Mol. Plant.

[14] Zhu Y.X., Du W.X., Fang X.Z., Zhang L.L., Jin C.W. (2020). Knockdown of BTS may provide a new strategy to improve cadmium-phytoremediation efficiency by improving iron status in plants. J. Hazard. Mater..

[15] Clemens S. (2001). Molecular mechanisms of plant metal tolerance and homeostasis. Planta.

[16] Lin Y.F., Aarts M.G. (2012). The molecular mechanism of zinc and cadmium stress response in plants. Cell Mol. Life Sci..

[17] Song W.Y., Martinoia E., Lee J., Kim D., Kim D.Y., Vogt E., Shim D., Choi K.S., Hwang I., Lee Y. (2004). A novel family of cys-rich membrane proteins mediates cadmium resistance in Arabidopsis. Plant Physiol..

[18] Morel M., Crouzet J., Gravot A., Auroy P., Leonhardt N., Vavasseur A., Richaud P. (2009). AtHMA3, a P1B-ATPase allowing Cd/Zn/Co/Pb vacuolar storage in Arabidopsis. Plant Physiol..

[19] Wong C.K.E., Cobbett C.S. (2009). HMA P-type ATPases are the major mechanism for root-to-shoot Cd translocation in Arabidopsis thaliana. N. Phytol..

[20] Hanikenne M., Talke I.N., Haydon M.J., Lanz C., Nolte A., Motte P., Kroymann J., Weigel D., Kramer U. (2008). Evolution of metal hyperaccumulation required cis-regulatory changes and triplication of HMA4. Nature.

[21] Hernandez L.E., Sobrino-Plata J., Montero-Palmero M.B., Carrasco-Gil S., Flores-Caceres M.L., Ortega-Villasante C., Escobar C. (2015). Contribution of glutathione to the control of cellular redox homeostasis under toxic metal and metalloid stress. J. Exp. Bot..

[22] Li F., Deng Y., Liu Y., Mai C., Xu Y., Wu J., Zheng X., Liang C., Wang J. (2023). Arabidopsis transcription factor WRKY45 confers cadmium tolerance via activating PCS1 and PCS2 expression. J. Hazard. Mater..

[23] Meyer Y., Buchanan B.B., Vignols F., Reichheld J.P. (2009). Thioredoxins and glutaredoxins: Unifying elements in redox biology. Annu. Rev. Genet..

[24] Ogata F.T., Branco V., Vale F.F., Coppo L. (2021). Glutaredoxin: Discovery, redox defense and much more. Redox Biol..

[25] Rouhier N., Couturier J., Jacquot J.P. (2006). Genome-wide analysis of plant glutaredoxin systems. J. Exp. Bot..

[26] Ziemann M., Bhave M., Zachgo S. (2009). Origin and diversification of land plant CC-type glutaredoxins. Genome Biol. Evol..

[27] Li S., Lauri A., Ziemann M., Busch A., Bhave M., Zachgo S. (2009). Nuclear activity of ROXY1, a glutaredoxin interacting with TGA factors, is required for petal development in Arabidopsis thaliana. Plant Cell.

[28] Murmu J., Bush M.J., DeLong C., Li S., Xu M., Khan M., Malcolmson C., Fobert P.R., Zachgo S., Hepworth S.R. (2010). Arabidopsis basic leucine-zipper transcription factors TGA9 and TGA10 interact with floral glutaredoxins ROXY1 and ROXY2 and are redundantly required for anther development. Plant Physiol..

[29] Li N., Muthreich M., Huang L.J., Thurow C., Sun T., Zhang Y., Gatz C. (2019). TGACG-BINDING FACTORs (TGAs) and TGA-interacting CC-type glutaredoxins modulate hyponastic growth in Arabidopsis thaliana. N. Phytol..

[30] Laporte D., Olate E., Salinas P., Salazar M., Jordana X., Holuigue L. (2012). Glutaredoxin GRXS13 plays a key role in protection against photooxidative stress in Arabidopsis. J. Exp. Bot..

[31] Ndamukong I., Abdallat A.A., Thurow C., Fode B., Zander M., Weigel R., Gatz C. (2007). SA-inducible Arabidopsis glutaredoxin interacts with TGA factors and suppresses JA-responsive PDF1.2 transcription. Plant J..

[32] Zander M., Chen S., Imkampe J., Thurow C., Gatz C. (2012). Repression of the Arabidopsis thaliana jasmonic acid/ethylene-induced defense pathway by TGA-interacting glutaredoxins depends on their C-terminal ALWL motif. Mol. Plant.

[33] Lai Z., Schluttenhofer C.M., Bhide K., Shreve J., Thimmapuram J., Lee S.Y., Yun D.J., Mengiste T. (2014). MED18 interaction with distinct transcription factors regulates multiple plant functions. Nat. Commun..

[34] D’Alessandro S., Ksas B., Havaux M. (2018). Decoding beta-Cyclocitral-Mediated Retrograde Signaling Reveals the Role of a Detoxification Response in Plant Tolerance to Photooxidative Stress. Plant Cell.

[35] Gutsche N., Thurow C., Zachgo S., Gatz C. (2015). Plant-specific CC-type glutaredoxins: Functions in developmental processes and stress responses. Biol. Chem..

[36] Couturier J., Didierjean C., Jacquot J.P., Rouhier N. (2010). Engineered mutated glutaredoxins mimicking peculiar plant class III glutaredoxins bind iron-sulfur centers and possess reductase activity. Biochem. Biophys. Res. Commun..

[37] Lysenko E.A., Klaus A.A., Kartashov A.V., Kusnetsov V.V. (2020). Specificity of Cd, Cu, and Fe effects on barley growth, metal contents in leaves and chloroplasts, and activities of photosystem I and photosystem II. Plant Physiol. Biochem..

[38] Baker N.R. (2008). Chlorophyll fluorescence: A probe of photosynthesis in vivo. Annu. Rev. Plant Biol..

[39] Alvarez M.E., Savoure A., Szabados L. (2022). Proline metabolism as regulatory hub. Trends Plant Sci..

[40] Kavi Kishor P.B., Sreenivasulu N. (2014). Is proline accumulation per se correlated with stress tolerance or is proline homeostasis a more critical issue?. Plant Cell Environ..

[41] Siripornadulsil S., Traina S., Verma D.P., Sayre R.T. (2002). Molecular mechanisms of proline-mediated tolerance to toxic heavy metals in transgenic microalgae. Plant Cell.

[42] Song W.Y., Choi K.S., Kim D.Y., Geisler M., Park J., Vincenzetti V., Schellenberg M., Kim S.H., Lim Y.P., Noh E.W. (2010). Arabidopsis PCR2 is a zinc exporter involved in both zinc extrusion and long-distance zinc transport. Plant Cell.

[43] Biswas M.S., Mano J. (2021). Lipid Peroxide-Derived Reactive Carbonyl Species as Mediators of Oxidative Stress and Signaling. Front. Plant Sci..

[44] Huang L.J., Li N., Thurow C., Wirtz M., Hell R., Gatz C. (2016). Ectopically expressed glutaredoxin ROXY19 negatively regulates the detoxification pathway in Arabidopsis thaliana. BMC Plant Biol..

[45] Lillig C.H., Berndt C., Holmgren A. (2008). Glutaredoxin systems. Biochim. Biophys. Acta.

[46] Rouhier N., Lemaire S.D., Jacquot J.P. (2008). The role of glutathione in photosynthetic organisms: Emerging functions for glutaredoxins and glutathionylation. Annu. Rev. Plant Biol..

[47] Couturier J., Jacquot J.P., Rouhier N. (2009). Evolution and diversity of glutaredoxins in photosynthetic organisms. Cell Mol. Life Sci..

[48] Lillig C.H., Berndt C. (2013). Glutaredoxins in thiol/disulfide exchange. Antioxid. Redox Signal.

[49] Couturier J., Przybyla-Toscano J., Roret T., Didierjean C., Rouhier N. (2015). The roles of glutaredoxins ligating Fe-S clusters: Sensing, transfer or repair functions?. Biochim. Biophys. Acta.

[50] Meyer Y., Belin C., Delorme-Hinoux V., Reichheld J.P., Riondet C. (2012). Thioredoxin and glutaredoxin systems in plants: Molecular mechanisms, crosstalks, and functional significance. Antioxid. Redox Signal.

[51] Chao D.Y., Silva A., Baxter I., Huang Y.S., Nordborg M., Danku J., Lahner B., Yakubova E., Salt D.E. (2012). Genome-wide association studies identify heavy metal ATPase3 as the primary determinant of natural variation in leaf cadmium in Arabidopsis thaliana. PLoS Genet..

[52] Keunen E., Peshev D., Vangronsveld J., Van Den Ende W., Cuypers A. (2013). Plant sugars are crucial players in the oxidative challenge during abiotic stress: Extending the traditional concept. Plant Cell Environ..

[53] Hu Y.Q., Liu S., Yuan H.M., Li J., Yan D.W., Zhang J.F., Lu Y.T. (2010). Functional comparison of catalase genes in the elimination of photorespiratory H2O2 using promoter- and 3’-untranslated region exchange experiments in the Arabidopsis cat2 photorespiratory mutant. Plant Cell Environ..

[54] Gao X., Yuan H.M., Hu Y.Q., Li J., Lu Y.T. (2014). Mutation of Arabidopsis CATALASE2 results in hyponastic leaves by changes of auxin levels. Plant Cell Environ..

[55] Davletova S., Rizhsky L., Liang H., Shengqiang Z., Oliver D.J., Coutu J., Shulaev V., Schlauch K., Mittler R. (2005). Cytosolic ascorbate peroxidase 1 is a central component of the reactive oxygen gene network of Arabidopsis. Plant Cell.

[56] Sunkar R., Kapoor A., Zhu J.-K. (2006). Posttranscriptional Induction of Two Cu/Zn Superoxide Dismutase Genes in Arabidopsis Is Mediated by Downregulation of miR398 and Important for Oxidative Stress Tolerance. Plant Cell.

[57] Dvořák P., Krasylenko Y., Ovečka M., Basheer J., Zapletalová V., Šamaj J., Takáč T. (2021). In vivo light-sheet microscopy resolves localisation patterns of *FSD1*, a superoxide dismutase with function in root development and osmoprotection. Plant Cell Environ..

[58] Mano J., Belles-Boix E., Babiychuk E., Inze D., Torii Y., Hiraoka E., Takimoto K., Slooten L., Asada K., Kushnir S. (2005). Protection against photooxidative injury of tobacco leaves by 2-alkenal reductase. Detoxication of lipid peroxide-derived reactive carbonyls. Plant Physiol..

[59] Fu Z.W., Ding F., Zhang B.L., Liu W.C., Huang Z.H., Fan S.H., Feng Y.R., Lu Y.T., Hua W. (2024). Hydrogen peroxide sulfenylates and inhibits the photorespiratory enzyme PGLP1 to modulate plant thermotolerance. Plant Commun..

[60] Zaffagnini M., Michelet L., Massot V., Trost P., Lemaire S.D. (2008). Biochemical characterization of glutaredoxins from Chlamydomonas reinhardtii reveals the unique properties of a chloroplastic CGFS-type glutaredoxin. J. Biol. Chem..

[61] Roach T., Baur T., Stoggl W., Krieger-Liszkay A. (2017). Chlamydomonas reinhardtii responding to high light: A role for 2-propenal (acrolein). Physiol. Plant.

[62] Zhu J., Wang W.S., Yan D.W., Hong L.W., Li T.T., Gao X., Yang Y.H., Ren F., Lu Y.T., Yuan T.T. (2023). CK2 promotes jasmonic acid signaling response by phosphorylating MYC2 in Arabidopsis. Nucleic Acids Res..

[63] Li T.T., Liu W.C., Wang F.F., Ma Q.B., Lu Y.T., Yuan T.T. (2018). SORTING NEXIN 1 Functions in Plant Salt Stress Tolerance Through Changes of NO Accumulation by Regulating NO Synthase-Like Activity. Front. Plant Sci..

[64] Ding F., Li F., Zhang B. (2022). A plastid-targeted heat shock cognate 70-kDa protein confers osmotic stress tolerance by enhancing ROS scavenging capability. Front. Plant Sci..

