# Supplementary material for: A CC-Type Glutaredoxins GRX480 Functions in Cadmium Tolerance by Maintaining Redox Homeostasis in Arabidopsis
Source: Int J Mol Sci. 2024 Oct 25;25(21):11455. doi: 10.3390/ijms252111455 (PMC11546484; doi:10.3390/ijms252111455)
Supplement: Supplementary file 1 [file ijms-25-11455-s001.zip › ijms-3216983-supplementary.pdf]

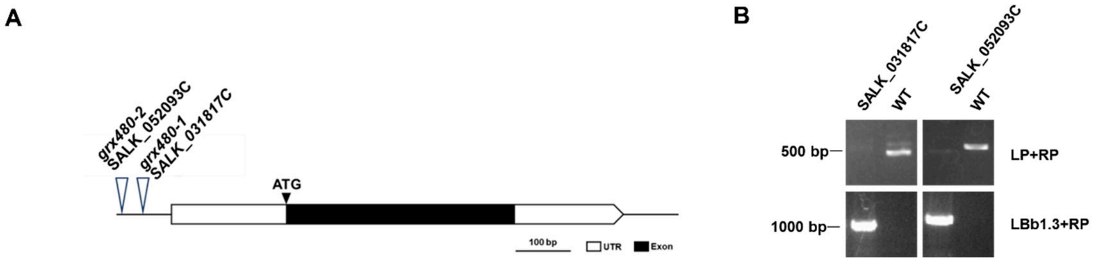

Supplemental Figure S1. Molecular characterization of *GRX480* mutants.

(A) Schematic representation of T-DNA insertion position of SALK_031871C and SALK_052093C. White boxes: UTR regions. Black boxes: exons. White triangle: T-DNA insertion. Black triangle: ATG start site. (B) PCR identified homozygous *GRX480* mutants.

Supplemental Table S1. List of all primers used in this study.

**Primer name**	**Sequence (5'-3')**
**Genomic DNA PCR**	
SALK_031817C LP	CAGAGCCCCAACTTCCTTAAG
SALK_031817C RP	CGGAAAATATCGAAAGTTTAGGC
SALK_052093.28.65.N LP	ACGTCTACCGACTTTTCCACC
SALK_052093.28.65.N RP	CTCCTCACCACATGACACATG
LBb1.3	ATTTTGCCGATTTCGGAAC
**Molecular Cloning**	
OE-GRX480 F	CAAATCTATCTCTCTCGAGATGCAAGGAACG ATTTCTTG
OE-GRX480 R	GGTCCTTATAATCCTCGAGTCACAACCACAG AGCCCCAA
**RT-qPCR**	
GRX480 F	GGCCGCTATCGCTTAAAACG
GRX480 R	GCAACATCCTCTCCGTCCAA
PCR2 F	TGATTGCTTTTCTGACTGCAA
PCR2 R	TGATTGCTTTTCTGACTGCAA
PDR8 F	CTACACTCTTCCTGAGAACCGAA
PDR8 R	TCATAGCCATCTCCGCAAACCC
HMA3 F	GGTTTATAGACAAATGCTCTC
HMA3 R	CAAAACATGCTGCTGACACA
CAT2 F	TGTTCCATACAGGAGCACCA
CAT2 R	AAGCCTATTTGGGGGATCAT
CAT3 F	AAGCCTATTTGGGGGATCAT
CAT3 R	AAGCCTATTTGGGGGATCAT
APX1 F	TTTCCACCCTGGAAGAGAGGAC
APX1 R	TCACAACCCTTGGTAGCATCAGG
CSD2 F	CGTCTTCTCATTCCTCCTTCC
CSD2 R	GGGTTGAAATGTGGTCCTGTT
FSD1 F	ACCGAAGACCAGATTACATA
FSD1 R	TGGCACTTACAGCTTCCCAA
GSH1 F	GATGGTTTAGAGCGCAGAGG
GSH1 R	TACGCTTTGTCCCCATTCTC
GSH2 F	ACCAACTGCATTCCCAGAAG
GSH2 R	GCCATCCAAGCTAACACGAT
PP2AA3 F	CGGTTGTGGAGAACATGATAC
PP2AA3 R	GAACCAAACACAATTCGTTGCTG
EIF4A1 F	TCATAGATCTGGTCCTTGAAACC
EIF4A1 R	GGCAGTCTCTTCGTGCTGAC
